# Salivary *LDOC1* is a gender-difference biomarker of oral squamous cell carcinoma

**DOI:** 10.7717/peerj.6732

**Published:** 2019-04-09

**Authors:** Chung-Ji Liu, Jen-Hao Chen, Shih-Min Hsia, Chiu-Chu Liao, Hui-Wen Chang, Tzong-Ming Shieh, Yin-Hwa Shih

**Affiliations:** 1 Department of Oral and Maxillofacial Surgery, MacKay Memorial Hospital, Taipei, Taiwan; 2 MacKay Medical College, Taipei, Taiwan; 3 Department of Medical Research, MacKay Memorial Hospital, Taipei, Taiwan; 4 Institute of Oral Biology, School of Dentistry, National Yang-Ming University, Taipei, Taiwan; 5 School of Dentistry, College of Dental Medicine, Kaohsiung Medical University, Kaohsiung, Taiwan; 6 Prosthodontics Department, Kaohsiung Medical University Hospital, Kaohsiung, Taiwan; 7 School of Nutrition and Health Sciences, College of Nutrition, Taipei Medical University, Taipei, Taiwan; 8 Department of Healthcare Administration, Asia University, Taichung, Taiwan; 9 Department of Dental Hygiene, College of Health Care, China Medical University, Taichung, Taiwan

**Keywords:** Salivary RNA, LDOC1, Biomarker, Oral squamous cell carcinoma

## Abstract

**Background:**

The X-linked tumor suppressor gene *LDOC1* is reported to be involved in oral cancer. The detection of biomarkers in salivary RNA is a non-invasive strategy for diagnosing many diseases. The aim of the present study was to investigate the potential of salivary *LDOC1* as a biomarker of oral cancer.

**Methods:**

We determined the expression levels of *LDOC1* in the saliva of oral squamous cell carcinoma (OSCC) subjects, and investigated its correlation with various clinicopathological characteristics. The expression levels of salivary *LDOC1* were detected in 53 OSCC subjects and 43 healthy controls using quantitative reverse transcription polymerase chain reaction (qRT-PCR) analysis. We used Fisher’s exact test to analyze the correlations between expression levels and clinicopathological characteristics.

**Results:**

Salivary *LDOC1* was significantly upregulated in females with OSCC (*p* = 0.0072), and significantly downregulated in males with OSCC (*p* = 0.0206). Eighty-nine percent of male OSCC subjects who smoked expressed low levels of *LDOC1*. OSCC cell lines derived from male OSCC subjects expressed low levels of *LDOC1*.

**Conclusions:**

A high level of salivary *LDOC1* expression is a biomarker of OSCC in females. A high percentage of male OSCC subjects who smoke express low levels of salivary *LDOC1*. A low level of salivary *LDOC1* expression is a biomarker of OSCC in males.

## Introduction

Oral cancer, which is a common malignancy with worldwide distribution, is two or three times more prevalent in males than in females (Rivera, 2015). The primary currently known lifestyle-related risk factors include smoking, drinking, and betel chewing, which are reportedly more common in males than in females (Muscat et al., 1996). There is evidence that males are exposed to more risk factors than women, which explains the higher incidence of oral cancer in males (Amtha et al., 2014; Chatterjee, Gupta & Bose, 2015). Oral squamous cell carcinoma (OSCC) accounts for more than 90% of oral cancers cases (Kao & Lim, 2015; Markopoulos, 2012). The late TNM stages are associated with poor 5-year survival rates. Therefore, early diagnosis and treatment are important with regard to oral cancer.

The diagnosis of oral cancer always performed by the dentists with visual examination and palpation of the oral mucosa (Shibahara, 2017). Saliva contains abundant RNA molecules (Fabryova & Celec, 2014; Li et al., 2006), which serve as potential diagnostic markers of cancer (Achour & Aguilo, 2018; Chen et al., 2018; Chi et al., 2018; Thiele et al., 2018; Yu et al., 2017). Moreover, saliva collection is a non-invasive method that can be used to detect risk-associated biomarkers for diseases, and is more acceptable to certain age groups—i.e., children and the elderly—than blood sampling (Liu & Duan, 2012; Zhang et al., 2016). There has been a surge of recent studies investigated in the potential use of salivary biomarkers to detect oral cancer (Sinevici & O’Sullivan, 2016). However, there are few studies of salivary X-link gene as biomarkers of oral cancer.

The Knudson’s two-hit mechanism describes the loss of function of tumor suppressor genes in both autosomal chromosome alleles. However, the loss of X-linked tumor suppressor gene function challenges the “two-hit inactivation” theory. A single genetic hit is sufficient to cause tumor development and prognosis. The aberrant expression of tumor suppressor genes on the X chromosome contributes to ovarian cancer (Yang-Feng et al., 1992), breast cancer (Sirchia et al., 2005), and prostate cancer (Stephan et al., 2002), and affects individuals that are more susceptible to cancer formation (Spatz, Borg & Feunteun, 2004).

Leucine Zipper, Down-regulated in Cancer-1 (*LDOC1*) is an X-linked tumor suppressor gene. It plays a crucial role in modulating cell proliferation via the NF*κ*B signaling pathway (Thoompumkal et al., 2016). The hypermethylation of the *LDOC1* promoter region and the downregulation of *LDOC1* in ovarian and cervical cancer cell lines (Buchholtz et al., 2014; Buchholtz et al., 2013) increase cell proliferation. Low *LDOC1* expression levels have been correlated with oral cancer caused by exposure to cigarette smoke (Lee et al., 2015). However, some studies have reported that *LDOC1* overexpression increases cell proliferation and is correlated with poor prognosis (Duzkale et al., 2011; Song et al., 2013). There have been few investigations into the effect of *LDOC1* expression in oral cancer. The role of *LDOC1* in oral cancer formation requires further clarification.

In the present study, we determined salivary *LDOC1* expression levels in oral squamous cell carcinoma (OSCC) subjects to investigate the potential of salivary *LDOC1* as a biomarker of oral cancer.

## Materials & Methods

### Clinical samples

The present study was approved by the Institutional Review Board (IRB) of MacKay Memorial Hospital (IRB number 17MMHIS053). Salivary samples from 53 OSCC patients and 43 normal subjects were kindly provided by the Department of Medical Research of MacKay Memorial Hospital. The saliva samples were collected from the OSCC subjects before surgery, and all samples were collected by spitting. The samples were immediately placed in DNase- and RNase-free tubes, and centrifuged at 2,000 g and 4 °C for 10 min to remove the cellular fraction. The supernatants were stored at −80 °C until required.

### Salivary RNA extraction

The salivary RNA was extracted from each sample using a PureLink RNA Kit (Thermo Fisher Scientific Inc., Waltham, MA, USA) to produce a 200 µL solution containing the RNA. We reduced the volume of these solutions to 10 µL each by ethanol precipitation. The RNA samples were then dissolved in nuclease-free water and stored at −80 °C until required.

### Quantitative reverse transcription polymerase chain reaction (qRT-PCR)

We used a random primer to reverse transcribe the RNA, as described in our previous study (Yin-Hua Shih et al., 2015). The design of the primers used for the two genes used in the qRT-PCR experiment (*LDOC1* and *GAPDH*) were taken from the Roche Universal ProbeLibrary website. The qRT-PCR was performed using a Roche LC-480 instrument (Roche, Basel, Switzerland), and the PCR regimen comprised: denaturing at 95 °C for 10 min, annealing at 60 °C for 30 s, extension at 72 °C for 1 s, and a total run of 60 cycles. The fold change in gene expression was compared between the OSCC and normal groups. The threshold of the statistical table was twofold higher or twofold lower than the average fold change in the normal group.

### Statistical analysis

We carried out the statistical analyses using Prism version 5 (GraphPad Software, San Diego, CA, USA) and SPSS version 12 (IBM Corporation, Quezon, Philippines). The data are expressed as the mean ± SD. Group differences were analyzed using the Mann–Whitney *U* test and Fisher’s exact test. A *p*-value of <0.05 was taken to indicate statistical significance.

## Results

### Clinicopathological characteristics of the 53 OSCC subjects

We enrolled 53 OSCC subjects and 43 normal subjects in the present study. Of the OSCC subjects: 20 were under 55 years old (37.7%) and 33 were over 55 (62.3%); the average age was 56.6; 27 were male and 26 were female; and 25 subjects smoked (47.1%) and 28 (52.9%) did not. The clinical characteristics are listed in Table 1; they include other factors such as drinking, betel chewing, tumor size, tumor differentiation, clinical stage, node metastasis, recurrence, radiotherapy, and tumor site. Two subjects experienced tumor recurrence and two subjects experienced node metastasis.

**Table 1:** Clinicopathological characteristics of 53 OSCC subjects.

**Feature**	**N(%)**
**Age, y**	
<55	20 (37.7)
≥55	33 (62.3)
**Sex**	
Male	27 (50.9)
Female	26 (49.1)
**Smoke**	
Yes	25 (47.1)
No	28 (52.9)
**Drink**	
Yes	16 (30.1)
No	37 (69.9)
**Betal chewing**	
Yes	21 (40.7)
No	32 (59.3)
**Tumor size**	
T1-T2	27 (50.9)
T3-T4	26 (49.1)
**Differentiation**	
Well	26 (49)
Moderate	22 (41.5)
Poor	5 (9.5)
**Clinical stage**	
I–II	20 (37.7)
III–IV	33 (62.3)
**Node metastasis**	
Yes	2 (3.7)
No	51 (96.3)
**Recurrence**	
Yes	2 (3.7)
No	51 (96.3)
**Radiotherapy**	
Yes	20 (37.7)
No	33 (62.3)
**Tumor site**	
Buccal	14 (26.4)
Tongue	19 (35.8)
Others	20 (37.8)

### *LDOC1* expression in the OSCC subjects differed between the genders

**Figure 1:**
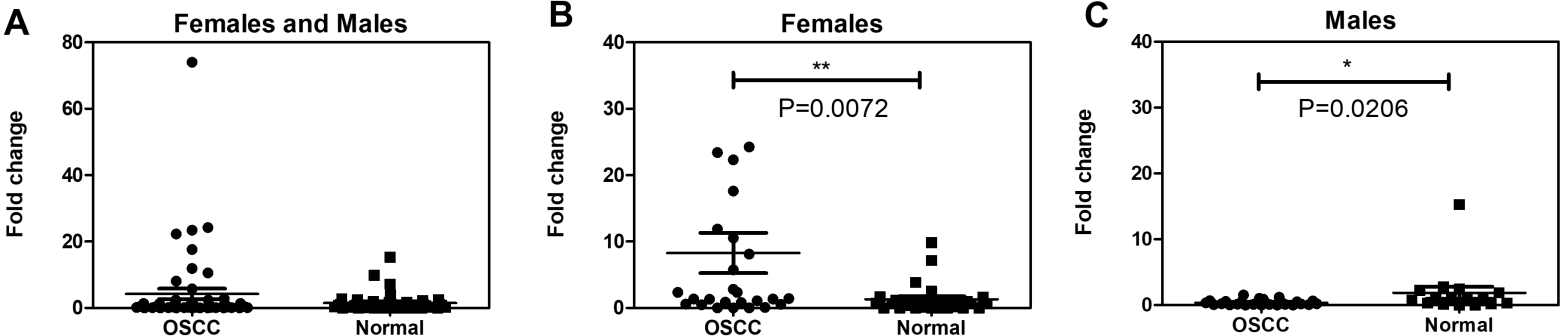
The salivary *LDOC1* expression in OSCC subjects and normal subjects. (A) The comparison of all subjects including female and male. (B) The comparison of female subjects. (C) The comparison of male subjects; males with OSCC *N* = 27, females with OSCC *N* = 26, normal male *N* = 16, normal female *N* = 27; Mann–Whitney *U* test.

The saliva samples were collected from the OSCC subjects by the Oral Maxillofacial Surgery Department doctors before the oral cancer operations. The saliva was immediately treated prior to storage according to the proper procedure mentioned above in the Materials & Methods section. We extracted the total RNA from the saliva and reverse transcribed it into complementary DNA (cDNA) using a random primer. The *LDOC1* cycle threshold (Ct) value was determined by the qRT-PCR machine. We compared the *LDOC1* expression levels between all the OSCC subjects and normal subjects, and found no significant differences (Fig. 1A). The subjects were then separated by gender and the *LDOC1* expression levels were compared. The *LDOC1* expression levels were significantly higher in females with OSCC (*p* = 0.0072) and significantly lower in males with OSCC (*p* = 0.0206). Figure 1 reveals significant differences in *LDOC1* expression levels between male and female OSCC subjects compared to those in normal subjects. The *LDOC1* expression levels showed gender-difference in the OSCC subjects.

### A high proportion of male OSCC subjects smoked and had low *LDOC1* expression levels

We statistically analyzed the correlations between *LDOC1* expression levels and various clinicopathological characteristics. There was no significant correlation between the clinicopathological characteristics and *LDOC1* expression levels in either the male or female OSCC subjects (Tables 2 and 3). However, among the 27 males with OSCC, the *LDOC1* expression levels were low in 24 (88.8%) cases, of whom more than 60% had a history of smoking (79.1%), a history of betel chewing (66.6%), had well-differentiated tumors (66.6%), were in clinical stages III–IV (65.4%), had no recurrence (83.3%), and no node metastasis (100%).

**Table 2:** The correlation between salivary *LDOC1* expression and clinicopathological characteristics in females with OSCC.

Feature	**LDOC1 expression**		*P* value
	**>2 fold N**	**Others N**	
**Age, y**			
<55	3	6	1
≥55	6	11	
**Smoke**			
Yes	2	1	0.27
No	7	16	
**Drink**			
Yes	1	0	0.35
No	8	17	
**Betal chewing**			
Yes	3	1	0.1
No	6	16	
**Tumor size**			
T1–T2	4	11	0.42
T3–T4	5	6	
**Differentiation**			
Well	4	4	0.37
Moderate	5	10	
Poor	0	3	
**Clinical stage**			
I–II	3	8	0.68
III–IV	6	9	
**Node metastasis**			
Yes	0	1	1
No	9	16	
**Recurrence**			
Yes	0	0	1
No	9	17	
**Radiotherapy**			
Yes	3	5	1
No	6	12	
**Tumor site**			
Buccal	3	1	0.12
Tongue	3	11	
Others (Gingiva, palate)	3	5	

**Notes.**

Fisher’s exact test, two tail; N, subject number; the definition of LDOC1 expression threshold described in ‘Materials and Methods’.

**Table 3:** The correlation between salivary *LDOC1* expression and clinicopathological characteristics in males with OSCC.

**Feature**	**LDOC1 expression**		*P* value
	**<2 fold N (%)**	**Others N**	
**Age, y**		
<55	13 (54.1)	1	0.5956
≥55	11 (45.9)	2	
**Smoke**			
Yes	19 (79.1)	3	1
No	5 (20.9)	0	
**Drink**			
Yes	12 (50)	3	0.2308
No	12 (50)	0	
**Betal chewing**			
Yes	16 (66.6)	1	0.535
No	8 (33.4)	2	
**Tumor size**			
T1–T2	10 (46.1)	2	0.5692
T3–T4	14 (53.9)	1	
**Differentiation**			
Well	16 (66.6)	2	1
Moderate	6 (25)	1	
Poor	2 (8.4)	0	
**Clinical stage**			
I–II	8 (33.4)	1	1	
III–IV	16 (66.6)	2
**Node metastasis**			
Yes	0 (0)	1	0.1111
No	24 (100)	2	
**Recurrence**			
Yes	2 (16.7)	0	1
No	22 (83.3)	3	
**Radiotherapy**			
Yes	12 (50)	0	0.2308
No	12 (50)	3	
**Tumor site**			
Buccal	9 (37.5)	1	1
Tongue	4 (16.6)	0	
Others			
(gingiva, palate, mouth floor)	11 (45.9)	2	

**Notes.**

Fisher’s exact test, two tail; N, subject number; the definition of the LDOC1 expression threshold described in ‘Materials and Methods’.

### The OSCC cell line derived from male OSCC subjects exhibited low *LDOC1* expression

**Figure 2:**
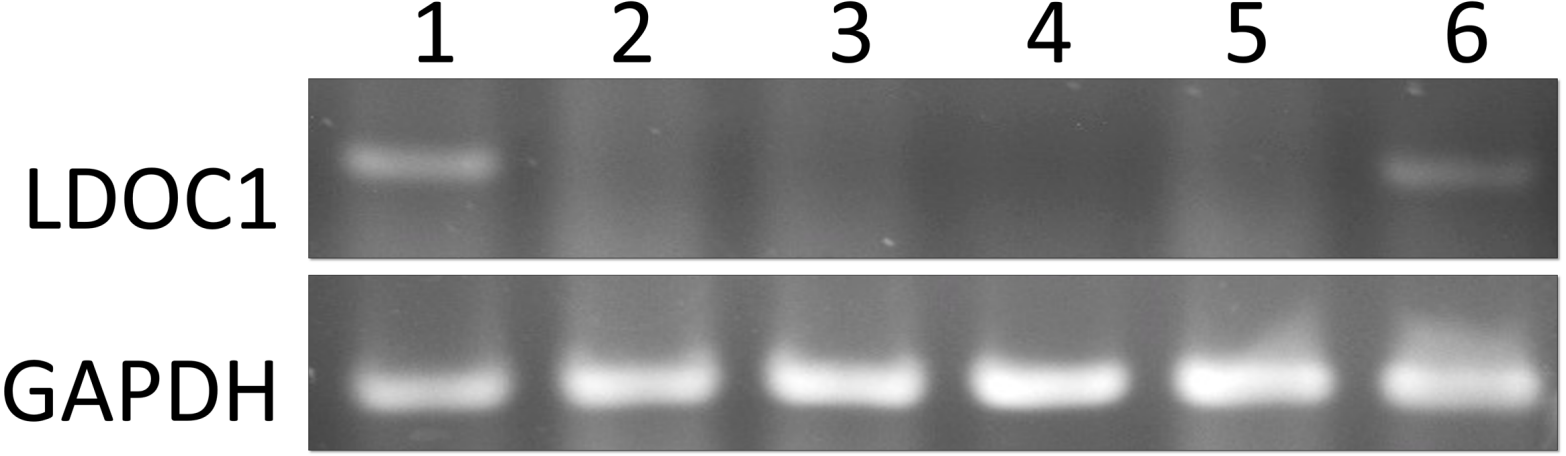
The *LDOC1* expression in cell lines. Number one to six represents different cell lines. 1. A549 male lung carcinoma; 2. HSC3 male tongue SCC; 3. Cal27 male tongue SCC; 4. OECM-1 male oral cavity SCC; 5. SAS female tongue SCC; 6. SG male human gingival epithelioid; SCC means squamous cell carcinoma.

We determined the levels of *LDOC1* expression in OSCC cell lines derived from OSCC subjects. Three of the cell lines derived from males with OSCC (HSC3, Cal27, and OECM-1), and one cell line derived from females with OSCC (SAS) had no *LDOC1* amplicons in the electrophoresis gel. We added a lung carcinoma cell line (A549) and a gingival cell line (SG), which were derived from male subjects, for comparison (Fig. 2). The low *LDOC1* expression levels in all the male OSCC cell lines provide secondary evidence that low *LDOC1* expression is correlated with male gender in OSCC.

## Discussion

Numerous studies have provided evidence to support the use of aberrant expression levels of tumor suppressor genes as a biomarker for the diagnosis of OSCC. To the best of our knowledge, the present study is the first investigation of the use of the expression levels of an X-linked gene in the saliva of OSCC subjects as a biomarker.

When it comes to enrollment of subjects, and collection of saliva. Males are two or three times more susceptible to oral cancer than females. Therefore, most clinical studies of oral cancer involve more male than female subjects. We aimed to investigate the relationship between the aberrant expression of an X-linked tumor suppressor gene and oral cancer. We deliberately attempted to enroll equal percentages of male and female OSCC subjects. As Table 1 shows, the final ratio of males to females was 1.2:1. Saliva collection in the present study was limited because it is difficult to collect saliva from patients suffering from severe oral cancer, especially those who have received both chemotherapy and radiotherapy and suffer from a dry mouth symptom. Therefore, most of the saliva specimens were collected from subjects without recurrence (96.3%) or node metastasis (96.3%).

Our qRT-PCR data revealed no significant difference between all the OSCC subjects and all the normal subjects (Fig. 1A). When we analyzed the *LDOC1* expression levels by gender, we found that there was a significant difference between male and female OSCC subjects compared to normal males and females (Figs. 1B and 1C). The literatures provided evidence of both upregulation and downregulation of *LDOC1* resulting in cancer. For future work, an *in vivo* investigation into how the difference in *LDOC1* expression levels between the genders contributes to the various mechanisms underlying tumorigenesis is required.

Several cancer studies have reported hypermethylation in the *LDOC1* promoter region (Buchholtz et al., 2014; Buchholtz et al., 2013; Lee et al., 2015), and that *LDOC1* silencing is associated with a history of cigarette smoking (Lee et al., 2013). In our data, smoking was more prevalent in the males than in the females (12% in the females with OSCC and 88% in the males with OSCC). However, smoking was not significantly associated with *LDOC1* expression in males with OSCC (Table 3). The association between *LDOC1* expression and smoking in OSCC remains unclear and is a potential future research topic.

We determined the *LDOC1* expression levels in OSCC cell lines derived from male and female OSCC subjects (Fig. 2). We have no information regarding the smoking habits of the OSCC subjects who donated the cells, but there was no evidence of *LDOC1* amplicon expression on the electrophoresis gel in any of the OSCC cell lines (three derived from males and one derived from females). This suggests that an absence of *LDOC1* expression in oral cells may be one of the causes of tumorigenesis.

## Conclusions

In summary, salivary *LDOC1* is a gender-difference biomarker of OSCC. Whether aberrant *LDOC1* expression contributes to OSCC is still unclear owing to a lack of direct evidence. Future *in vitro* and *in vivo* studies are required to determine how gender-specific *LDOC1* expression contributes to oral cancer.

##  Supplemental Information

10.7717/peerj.6732/supp-1 Supplemental Information 1 Clinical characteristics and Ct value of the qPCR

10.7717/peerj.6732/supp-2 Supplemental Information 2 The raw data of the gel electrophoresis The left side of the marker is the amplicons of GAPDH, and the right side is the amplicons of LDOC1. Number 1 to 6 indicates six different cell lines. Neg means negative control of the PCR. The arrow indicates the actual size of the LDOC1 amplicons.

## Additional Information and Declarations

### Data Availability

The following information was supplied regarding data availability: The raw data of clinicopathological characteristics and the Ct value of LDOC1 are available in the Supplemental Files.
