# Supplementary figures and images for: Salivary LDOC1 is a gender-difference biomarker of oral squamous cell carcinoma
Source: PeerJ. 2019 Apr 9;7:e6732. doi: 10.7717/peerj.6732 (PMC6461027; doi:10.7717/peerj.6732)

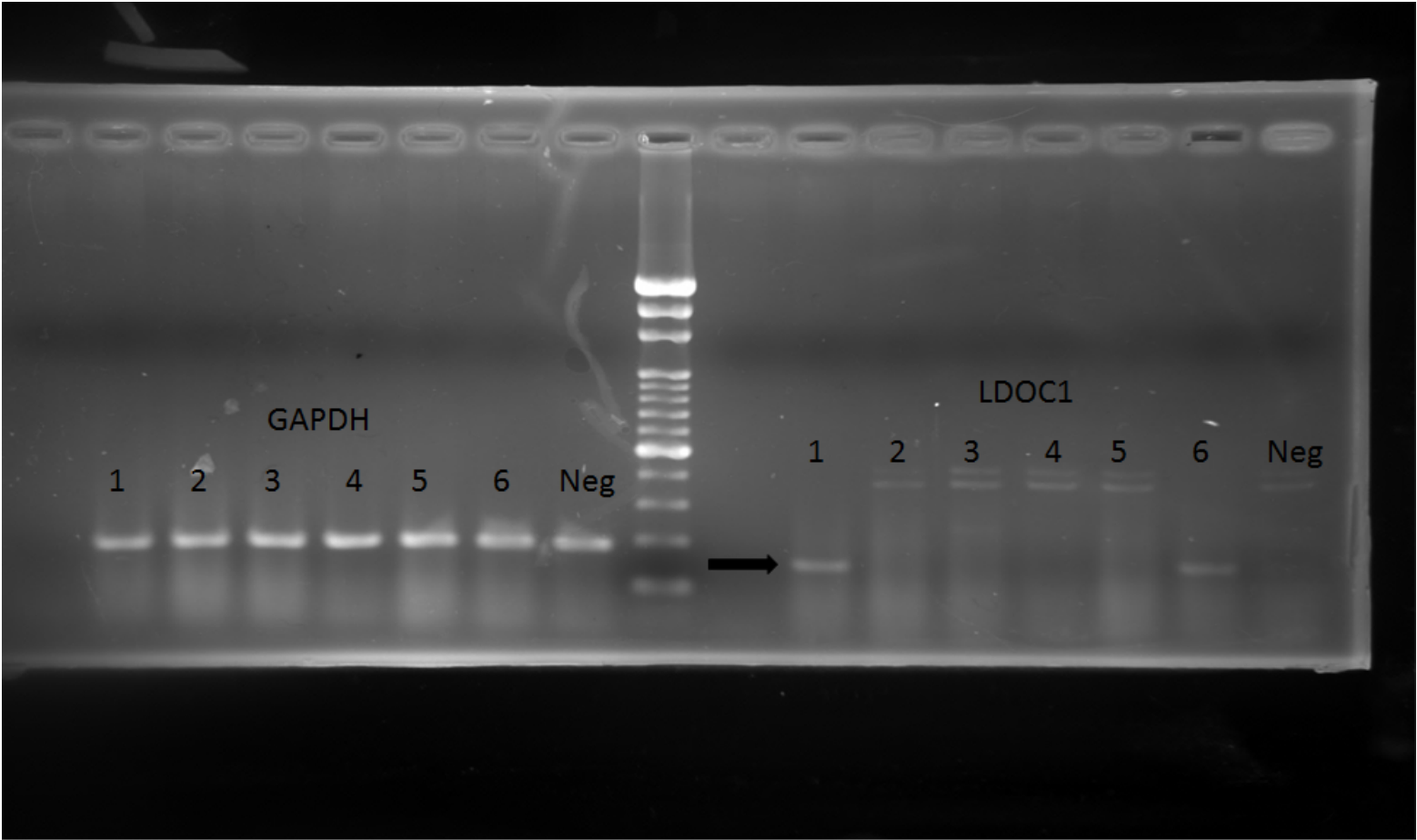

Supplement: Supplemental Information 2 — The left side of the marker is the amplicons of GAPDH, and the right side is the amplicons of LDOC1. Number 1 to 6 indicates six different cell lines. Neg means negative control of the PCR. The arrow indicates the actual size of the LDOC1 amplicons. [file peerj-07-6732-s002.pdf]
